# Integrated computational analysis prioritizes candidate targets and pathways linking ochratoxin A exposure to hepatocellular carcinoma

**DOI:** 10.1371/journal.pone.0357594

**Published:** 2026-08-31

**Authors:** Shili Yang, Huaiquan Liu, Haiyang Kou, Lingyan Lai, Xinyan Zhang, Yunling Xu, Yu Sun, Bo Chen

**Affiliations:** Guizhou University of Traditional Chinese Medicine, Guiyang, Guizhou Province, China; Ankara University: Ankara Universitesi, TÜRKIYE

## Abstract

Ochratoxin A (OTA), a food-borne mycotoxin, has been implicated in hepatotoxicity and potential carcinogenic processes, yet the molecular links between OTA exposure and hepatocellular carcinoma (HCC) remain incompletely understood. This study used an integrated computational workflow to prioritize candidate targets and pathways potentially linking OTA exposure with HCC. OTA-related and HCC-related targets were collected from public databases, intersected, and subjected to functional enrichment analysis. Transcriptomic data from the GSE36376 discovery dataset were analyzed to identify differentially expressed genes, followed by LASSO and SVM-RFE feature selection, immune-cell deconvolution, molecular docking, and molecular dynamics simulation. A total of 214 overlapping OTA-HCC-associated targets were identified and were enriched in pathways related to signal transduction, apoptosis, metabolism, and immune regulation. In GSE36376, 443 differentially expressed genes were identified using p < 0.05 and |log2 fold change| > 1, and overlap analysis yielded 13 shared target genes. Five candidate targets, CYP3A4, KIFC1, AKR1C3, CA2, and TTR, were further prioritized. KIFC1 and AKR1C3 were upregulated in HCC samples, whereas CYP3A4, CA2, and TTR were downregulated. These genes showed apparent discriminatory ability within the discovery dataset, with AUC values ranging from 0.866 to 0.958. Molecular docking predicted favorable OTA-target interactions, with docking energies ranging from −7.4 to −10.8 kcal/mol. CYP3A4 showed the lowest predicted docking energy (−10.8 kcal/mol) and was further evaluated by molecular dynamics simulation, with a protein-fitted OTA RMSD of 1.435 ± 0.097 nm and complex Rg of 2.308 ± 0.010 nm during the equilibrated 20–100 ns trajectory. Overall, this study provides a reproducible hypothesis-generating framework for exploring potential metabolic, genomic-instability-related, and immune-microenvironment links between OTA exposure and HCC. Future validation in independent datasets and experimental models will be important to further assess the biological relevance of these candidate targets and pathways.

## Introduction

Hepatocellular carcinoma (HCC), the most common primary liver malignancy, is a leading cause of cancer-related mortality worldwide and accounts for a substantial global disease burden [1]. More than half of the world’s new annual liver cancer cases occur in China, where patient prognosis is generally poor, with an overall 5-year survival rate of only about 10% [2]. Therefore, research on the prevention and treatment of HCC holds significant practical and clinical value. The etiology of HCC is complex. The main pathogenic factors in the Chinese population include chronic Hepatitis B virus (HBV) infection and aflatoxin exposure. In Japan, Hepatitis C virus (HCV) infection is predominant, while alcohol and metabolic disorders are more common in Western populations [3]. Current research suggests that HCC development results from the interaction of multiple factors, involving complex interplay among genetic susceptibility, environmental toxins, and metabolic dysregulation [4]. Among these, Ochratoxin A (OTA), a mycotoxin widely present worldwide, is a secondary metabolite produced by Penicillium and Aspergillus fungi [5] and can be detected in various countries. Due to climatic conditions or improper food storage, OTA may contaminate grains, meat, fruits, wine, beer, coffee, and other foods, and has been associated with several toxic effects, particularly nephrotoxicity and hepatotoxicity [6]. Currently, epidemiological and preclinical studies increasingly indicate a close association between OTA and HCC [7]. Although previous studies have provided background information on the potential association between OTA exposure and HCC, the underlying molecular mechanisms remain incompletely understood. Traditional toxicology studies are often limited to the analysis of single targets or pathways, showing certain limitations when explaining diversified toxicity regulation networks. With the development of computational toxicology, network-based system toxicology has emerged as a useful approach for integrating compound-target-pathway-disease relationships and exploring potential toxicological cascades [7]. Machine learning methods can further support candidate feature prioritization and pattern recognition in biomedical datasets [8]. Previous HCC studies have reported alterations in hepatic xenobiotic metabolism, mitotic regulation, reductase-related pathways, carbonic anhydrase activity, and liver-derived transport processes, including studies involving CYP3A4, KIFC1, AKR1C3, CA2, and TTR, thereby providing biological context for interpreting computationally prioritized genes in these functional categories [9–13]. In the present study, we integrated network toxicology, machine learning, immune infiltration estimation, molecular docking, and molecular dynamics simulation to explore potential molecular associations between OTA exposure and HCC. This workflow was designed to prioritize OTA-HCC-associated candidate targets and pathways, evaluate their expression patterns and immune-cell associations, and further characterize potential ligand-protein interactions through structural modeling.

## Materials and methods

### Preparation of OTA compound information

The Simplified Molecular Input Line Entry System (SMILES) of OTA was searched and saved via the PubChem database (https://pubchem.ncbi.nlm.nih.gov), and its 2D structure was downloaded for later use.

### Analysis of OTA-related toxicity targets

OTA-related toxicity targets were retrieved from the ChEMBL database (https://www.ebi.ac.uk/chembl), the SEA database (https://sea.bkslab.org/), and the SwissTargetPrediction database (https://www.swisstargetprediction.ch), with the species limited to “Homo sapiens.” After removing duplicate targets from the search results, the Uniprot database (https://www.uniprot.org/) was used for gene name standardization. The OTA-related toxicity target genes were then organized. Finally, the intersection of target genes from the three databases was taken using a Venn diagram, and visual analysis was completed.

### Analysis of HCC-related disease targets

Using “hepatocellular carcinoma” as the search keyword in the GeneCards database (https://www.genecards.org) and the OMIM database (https://omim.org), target genes with a Relevance score > 5 in the GeneCards database were screened, and related target genes from the OMIM database were extracted. Then, the intersection of the two types of target genes was taken using a Venn diagram to determine HCC-related disease targets.

### Intersection of OTA target genes and HCC-related genes

The previously obtained OTA-related toxicity target genes and HCC-related target genes were intersected using a Venn diagram to obtain the intersecting genes between them.

### GO and KEGG enrichment analyses

The screened intersecting genes were converted from gene symbols to Entrez IDs based on the human gene annotation database org.Hs.e.g.,db. Invalid genes without matching IDs were filtered out to obtain the analysis gene set. Using R packages such as clusterProfiler and enrichplot, GO and KEGG enrichment analyses were sequentially performed on the analysis gene set. GO enrichment analysis was conducted independently for three ontologies: Biological Process (BP), Cellular Component (CC), and Molecular Function (MF). The thresholds for both p-value and q-value were set at 0.05 to screen significantly enriched terms, and bubble charts were used to visualize the analysis results. KEGG pathway enrichment analysis was performed with the human (hsa) species background. The screening thresholds were set at p < 0.05 and adjusted p-value (p.adjust) < 0.05 to obtain significantly enriched pathways. A dot plot was also drawn to visually display the pathway enrichment characteristics.

### Download and organization of GEO data for HCC

“Hepatocellular Carcinoma” was entered in the GEO database (https://www.ncbi.nlm.nih.gov/gds) to search for related diseases, with conditions limited to “Series,” “expression profiling by array,” and “Homo sapiens.” The probe matrix file and platform file (GSE36376) related to HCC in this database were downloaded. Genes with p < 0.05 and |log2 fold change| > 1 were defined as significantly differentially expressed genes and visualized. This combined threshold was used to identify genes with both statistical significance and biologically meaningful expression changes, thereby reducing the inclusion of genes with very small but statistically significant differences. The resulting DEGs were then cross-referenced with the OTA-HCC-associated target set obtained from the network toxicology analysis. Genes shared by both datasets were retained as overlapping target genes for subsequent machine-learning-based prioritization.

### Screening of candidate targets using machine learning algorithms

This study employed two machine learning algorithms, Least Absolute Shrinkage and Selection Operator (LASSO) and Support Vector Machine-Recursive Feature Elimination (SVM-RFE), to prioritize candidate target genes. LASSO logistic regression was performed using the glmnet package, with alpha = 1 and family = “binomial”. The penalty parameter lambda was selected by 10-fold cross-validation, and genes with non-zero coefficients at lambda.min were retained. SVM-RFE was performed to rank features using support vector machine-based recursive feature elimination, and cross-validation was used to estimate the classification error for different feature numbers. The final candidate targets were defined as the overlapping genes selected by LASSO and SVM-RFE. Because feature selection and ROC analysis were performed using the same GSE36376 discovery dataset, the ROC curves were interpreted as internal performance estimates rather than independent external validation.

### Internal discriminatory performance assessment of candidate genes

The expression levels of the five candidate genes were compared between HCC and control samples in the GSE36376 discovery dataset. ROC curves were plotted using the pROC package to assess the apparent discriminatory ability of each candidate gene within the same dataset. Because the same discovery dataset was used for candidate gene prioritization and ROC analysis, the ROC curves were interpreted as internal performance estimates rather than independent external validation.

### Immune infiltration analysis

Immune-cell fractions were estimated using a CIBERSORT-like support vector regression deconvolution approach with an LM22-format 22 immune-cell reference signature matrix. The GSE36376 expression matrix was used as the mixture file. Quantile normalization was enabled, and 1,000 permutations were used to estimate empirical deconvolution P values. The relative fractions of immune-cell subsets were compared between HCC and control samples using the Wilcoxon rank-sum test. Spearman correlation analysis was used to examine associations between prioritized gene expression and estimated immune-cell fractions. The CIBERSORT results were interpreted as computational estimates from bulk transcriptomic data rather than direct measurements of immune-cell abundance.

### Molecular docking

The three-dimensional structures of the candidate target proteins were obtained from the Protein Data Bank (PDB), and the molecular structure of OTA was obtained from PubChem. Protein and ligand structures were prepared using Open Babel, AutoDockTools-1.5.6, and PyMOL 3.2. Molecular docking was performed using AutoDock Vina with default search parameters unless otherwise specified. The docking grid box size was set to 40 × 40 × 40 Å for all five candidate proteins. The grid box centers were set as follows: CYP3A4, (−19.161, −23.997, −13.938); KIFC1, (50.492, 23.003, 103.496); AKR1C3, (−5.909, −18.824, 33.216); CA2, (−9.709, −1.666, 15.002); and TTR, (21.066, 30.116, 48.963). The energy_range and num_modes parameters were set to 5 and 30, respectively. Docking poses were ranked according to the predicted binding energy, and the lowest-energy poses were used for visualization.

### Molecular dynamics simulation

GROMACS software was used to perform molecular dynamics simulation on the protein receptor-ligand complex [14,15]. After preprocessing the protein structure, the CHARMM36 force field and the TIP3P water model were selected. The topology file was modified to define ligand atom types. System construction used a cubic simulation box (minimum distance of molecule to box wall 1.0 nm). After solvation using the spc216 water box, Na ⁺ /Cl− ions were added to neutralize the system charge. Energy minimization was completed using the steepest descent method to eliminate unreasonable atomic contacts. Subsequently, 300 K NVT (isothermal-isochoric) equilibration and 1 bar NPT (isothermal-isobaric) equilibration were performed sequentially to stabilize system temperature and density. Using the equilibrated system as the starting point, 100 ns of main MD production sampling was conducted. Trajectories were processed to remove periodic boundary artifacts and centered on the protein for alignment. Subsequent analyses included: protein-ligand Root Mean Square Deviation (RMSD) to assess binding stability; Root Mean Square Fluctuation (RMSF) to analyze atomic/residue flexibility; Radius of Gyration (Rg) to reflect protein compactness; Solvent Accessible Surface Area (SASA) to quantify surface exposure; hydrogen bond number statistics to indicate interaction strength. Two-dimensional and three-dimensional free energy landscapes were generated using gmx sham combined with Python scripts (relying on matplotlib and scipy libraries) to analyze system conformational distribution and energy barriers.

## Results

### Network toxicological analysis of potential OTA-HCC-associated targets

**Fig 1:**
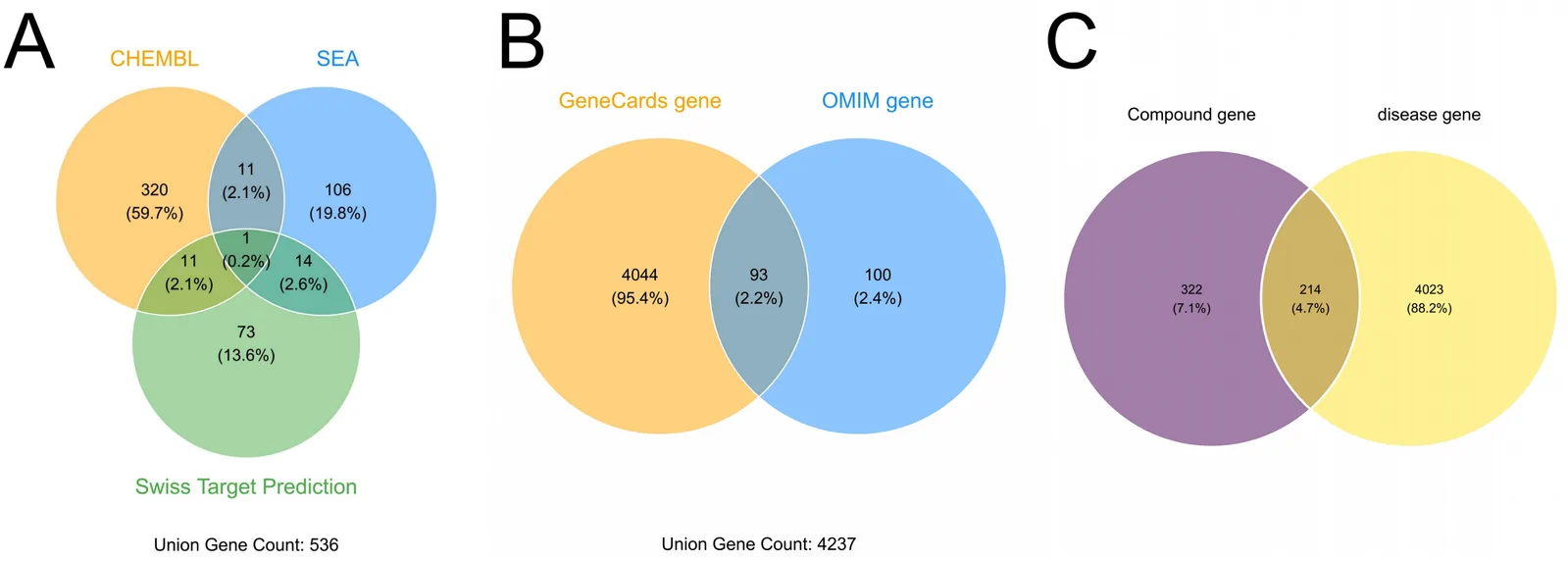
Determination of potential OTA-HCC-associated targets. (A) Screening of OTA target genes. (B) Screening of HCC target genes. (C) Intersection of OTA and HCC target genes.

A total of 536 OTA-related target genes were screened from the ChEMBL, SEA, and SwissTargetPrediction databases, as shown in Fig 1A. A total of 4237 HCC target genes were obtained from the GeneCards and OMIM databases, as shown in Fig 1B. After removing duplicate target genes, the intersection of the two types of target genes yielded 214 genes, which were used as potential OTA-HCC-associated targets, as shown in Fig 1C.

### GO enrichment analysis of OTA-HCC-associated targets

**Fig 2:**
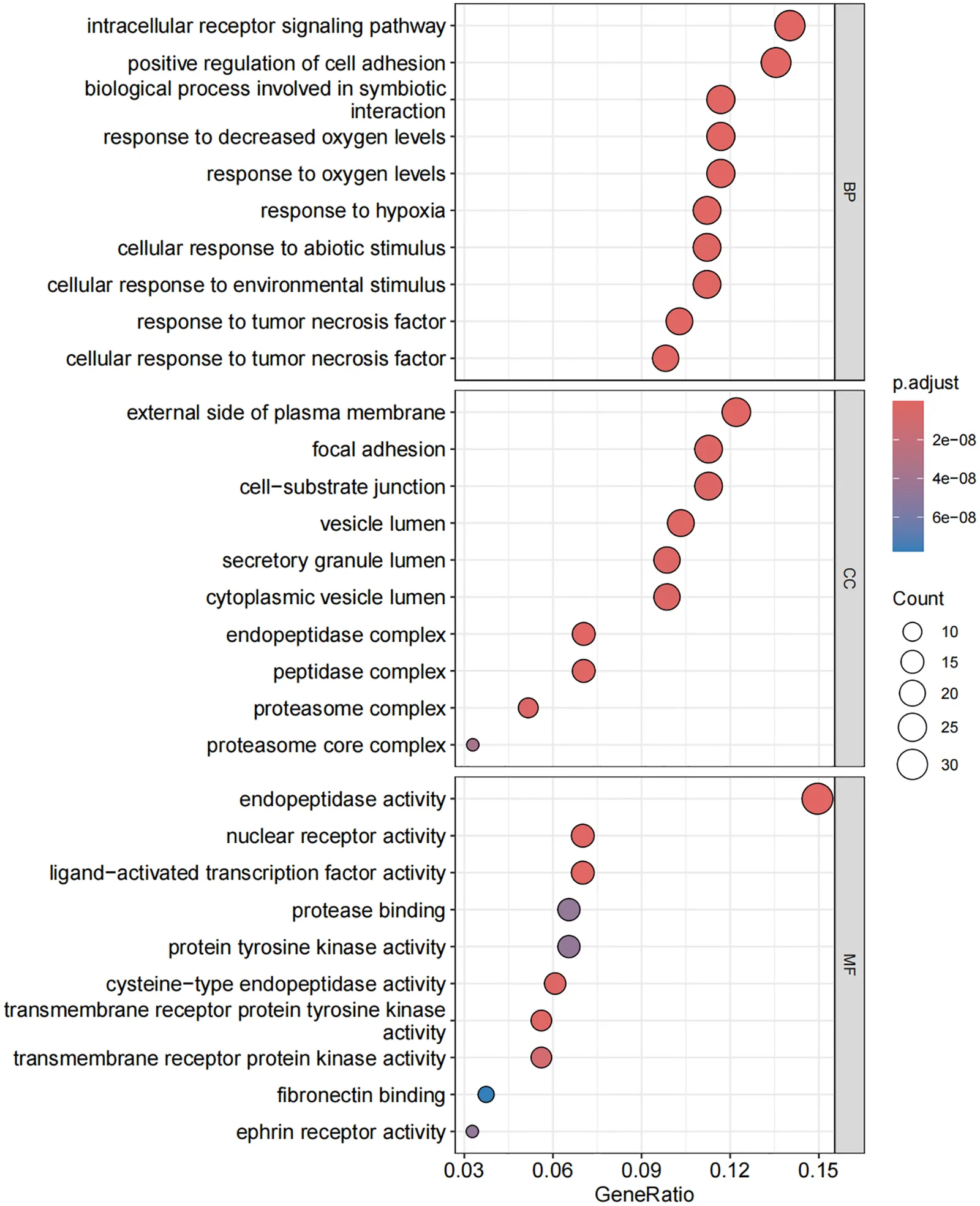
GO enrichment bubble chart.

GO enrichment analysis was performed based on the 214 intersecting genes of OTA and HCC, with q-value < 0.05. A total of 1799 statistically significant entries were obtained, including 1555 Biological Processes (BP), 83 Cellular Components (CC), and 161 Molecular Functions (MF). The top 10 entries from each category were extracted to draw bar charts for visualization. Redder colors in the chart represent lower adjusted p-values, indicating higher GO enrichment levels, as shown in Fig 2. The BP results of GO enrichment showed that intracellular receptor signaling pathway (GO:0030522), positive regulation of cell adhesion (GO:0045785), biological process involved in symbiotic interaction (GO:0044403), and other terms were among the enriched biological processes. In the CC category, external side of plasma membrane (GO:0009897), focal adhesion (GO:0005925), cell-substrate junction (GO:0030055), and other terms were enriched. In the MF category, endopeptidase activity (GO:0004175), nuclear receptor activity (GO:0004879), ligand-activated transcription factor activity (GO:0098531), and other terms were enriched.

### KEGG enrichment analysis of OTA-HCC-associated targets

**Fig 3:**
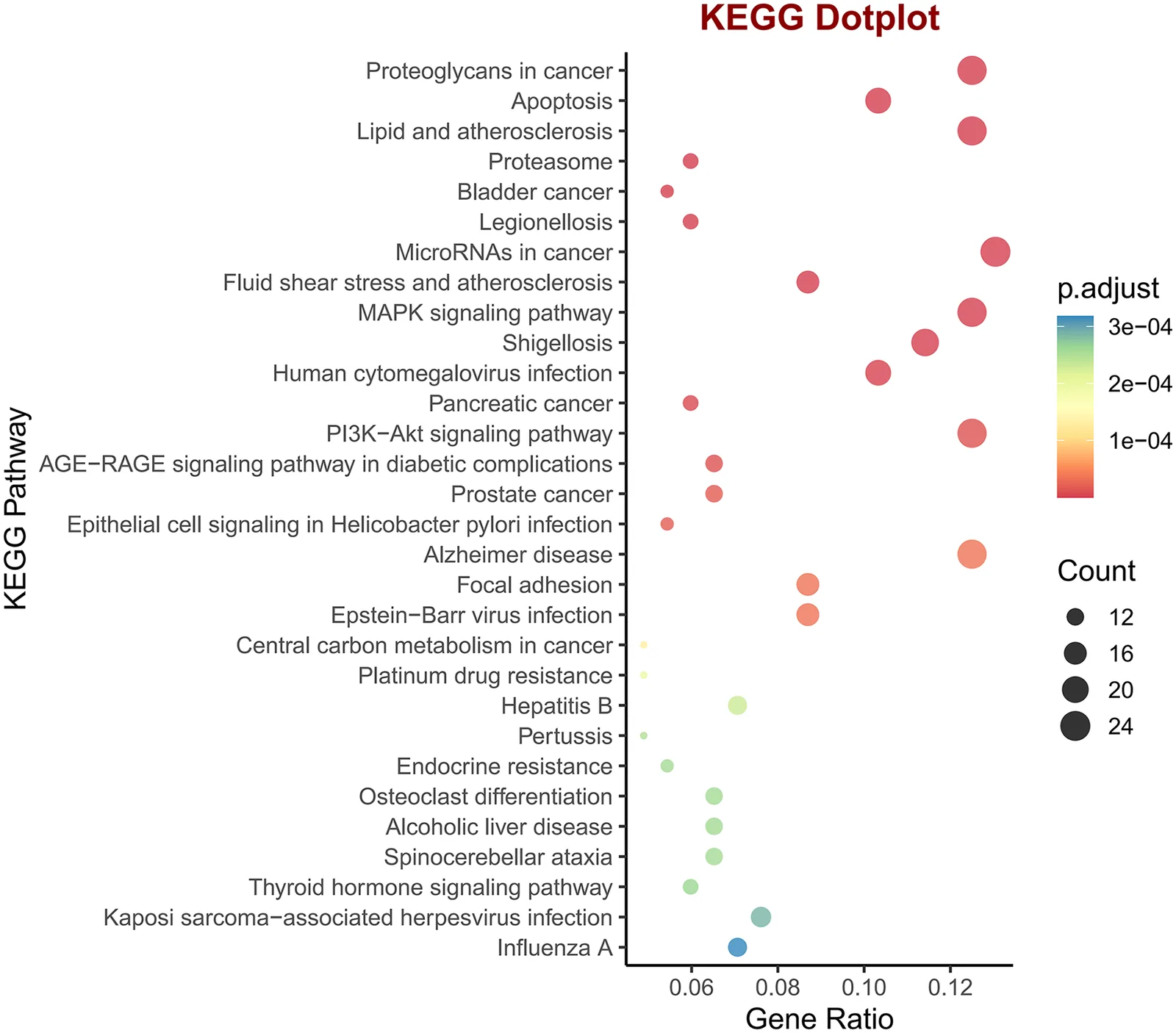
KEGG enrichment bubble chart.

KEGG enrichment analysis was performed based on the 214 intersecting genes of OTA and HCC, with q-value < 0.05. A total of 144 statistically significant pathways were obtained. The 30 enrichment pathways with the highest gene counts were extracted and a dot plot was drawn for visualization, as shown in Fig 3. These included Proteoglycans in cancer, Apoptosis, and Lipid and atherosclerosis, among others.

### GEO differential gene expression and target gene determination

**Fig 4:**
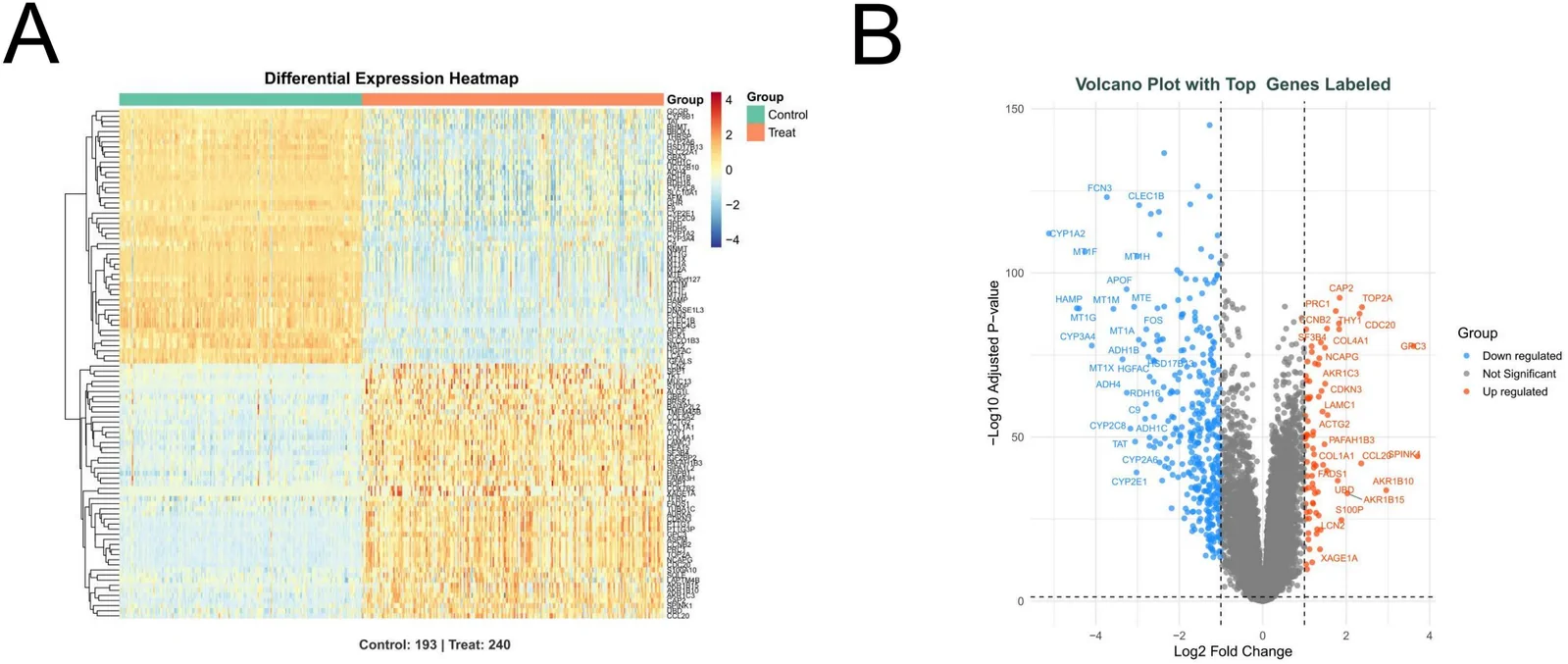
Visualization of differentially expressed genes. (A) Heatmap of differentially expressed genes. Redder color represents higher gene expression, bluer color represents lower gene expression. (B) Volcano plot of differentially expressed genes. Red represents high expression in disease, blue represents low expression in disease.

**Fig 5:**
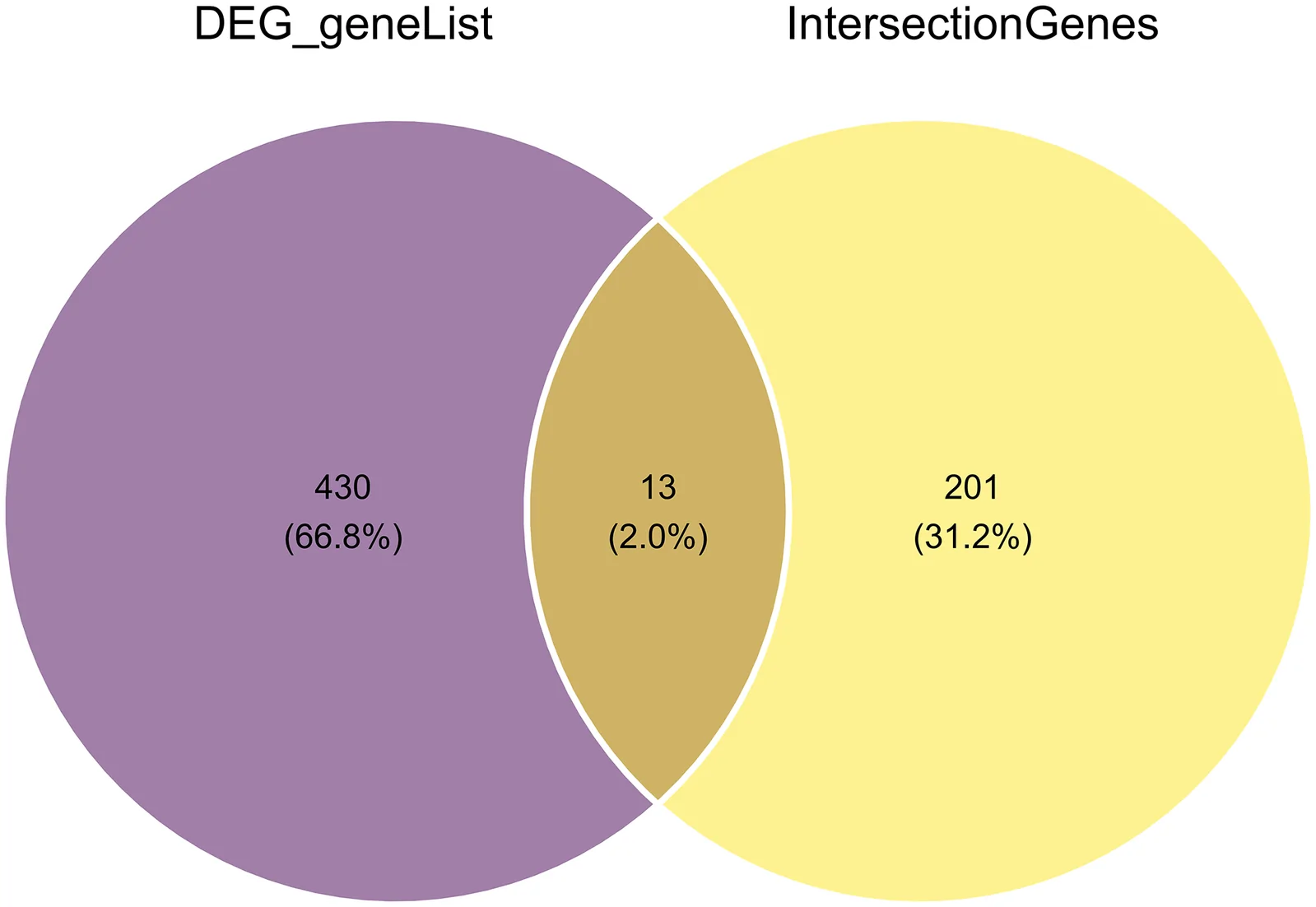
Overlap analysis identifying 13 shared target genes between differentially expressed genes and OTA-HCC-associated targets.

In the differential analysis based on the GSE36376 dataset, the limma package of R version 4.4.3 was used to complete data normalization and identification of differentially expressed genes (DEGs). The screening criteria were set as p < 0.05 and absolute log2 fold change greater than 1 (|log2 fold change| > 1). A total of 443 DEGs were obtained, and a heatmap and a differential expression volcano plot were drawn, as shown in Fig 4A and 4B, respectively. To focus on targets supported by both transcriptomic dysregulation and network toxicology prediction, the 443 DEGs were cross-referenced with the 214 OTA-HCC-associated targets. This overlap analysis identified 13 shared target genes, including ESR1, CYP3A4, KIFC1, AKR1C3, CA2, EGR1, SLC27A5, LMNA, PON1, PLG, TTR, MMP9, and AHSG, as shown in Fig 5.

### Prioritization of candidate targets using machine learning

**Fig 6:**
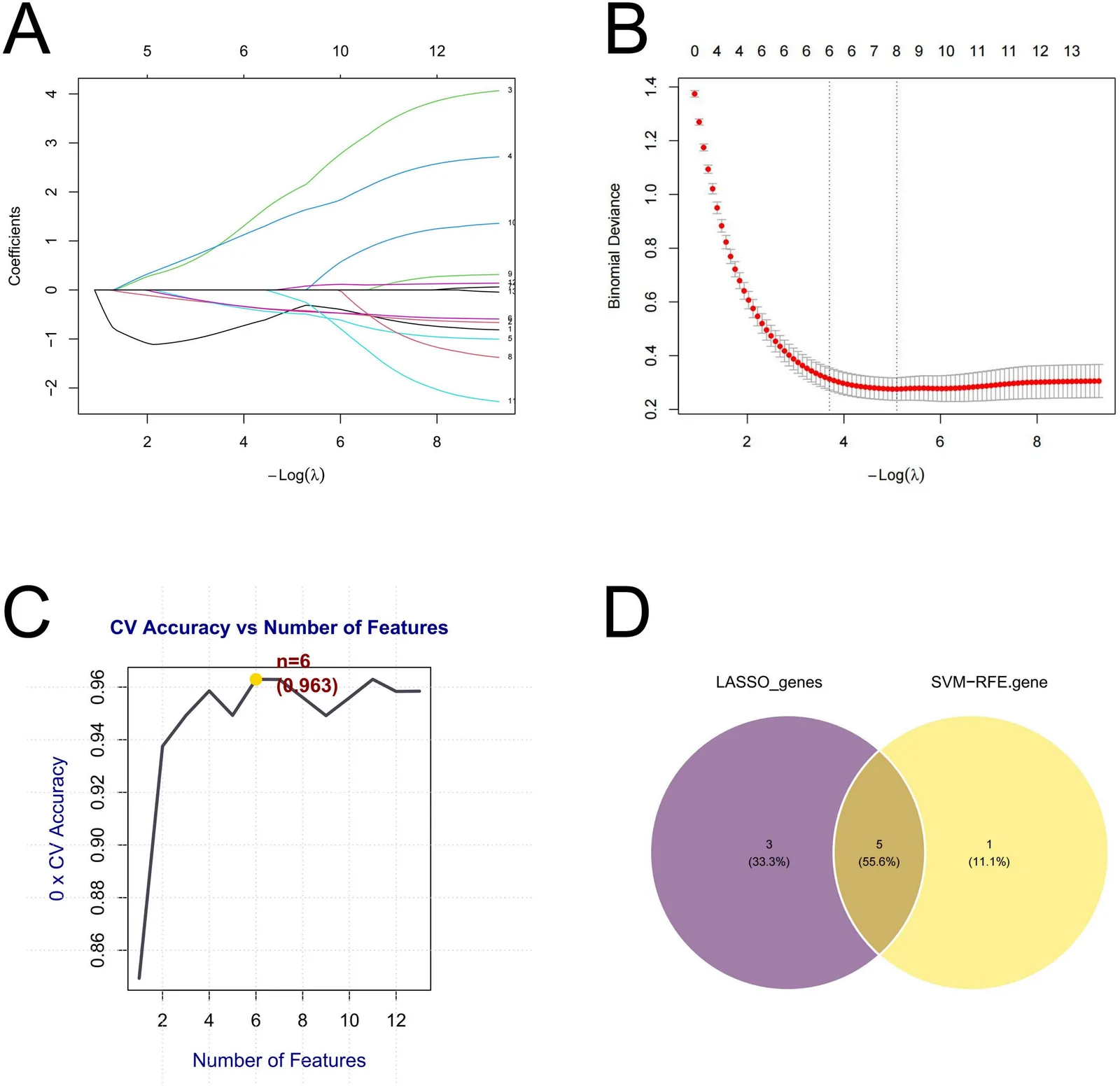
Machine learning-based prioritization of candidate targets. (A, B) LASSO logistic regression technique. The horizontal axis represents the number of model genes corresponding to different λ values. At the minimum λ value, 8 genes were identified. (C) SVM-RFE algorithm prioritized candidate genes, where six candidate genes were selected. (D) Venn diagram shows the common intersection of LASSO and SVM-RFE algorithms, prioritizing the final five candidate targets.

Subsequently, LASSO and SVM-RFE algorithms were used to prioritize candidate genes from the 13 target genes obtained from the intersection of DEGs and OTA-HCC-associated genes. First, through the parameter tuning process of LASSO logistic regression, cross-validation was used to evaluate the penalty parameter λ, combined with the coefficient path, as shown in Fig 6A, and the binomial deviance curve, as shown in Fig 6B, prioritizing 8 potential candidate targets. Then, the SVM-RFE algorithm was used to further narrow down the scope. Combining the relationship curve between the number of features and cross-validation accuracy, when the number of features was 6, the cross-validation accuracy was higher, as shown in Fig 6C, selecting 6 candidate targets. Finally, by taking the intersection of the screening results of the two methods using a Venn diagram, as shown in Fig 6D, five candidate targets were prioritized: CYP3A4, KIFC1, AKR1C3, CA2, and TTR.

### Expression patterns and internal discriminatory performance of the five candidate targets

**Fig 7:**
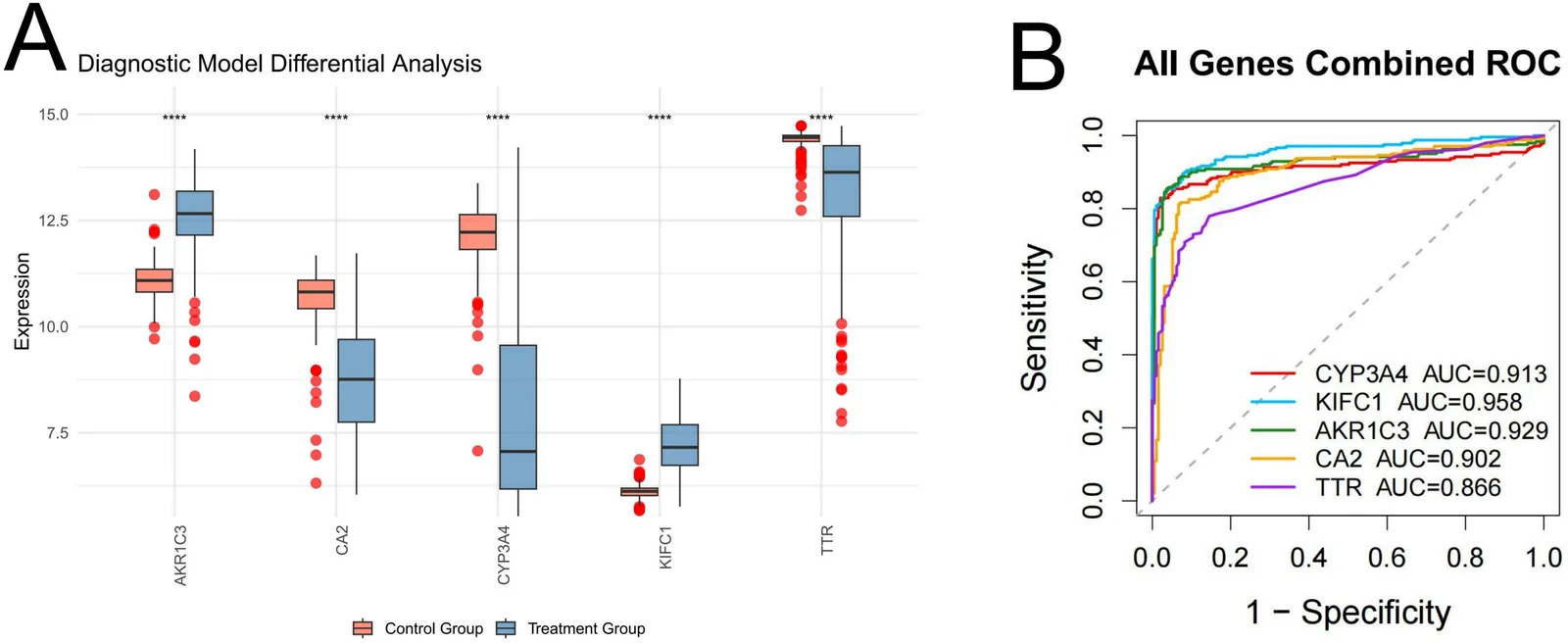
RNA-seq analysis of candidate gene expression and internal discriminatory performance in the GSE36376 discovery dataset. (A) Expression of the five candidate targets in HCC tissue samples versus control tissue samples. (B) ROC curves showing the apparent discriminatory ability of the five candidate targets within the same discovery dataset.

In the GSE36376 discovery dataset, the five prioritized candidate genes showed differential expression between HCC and control samples. KIFC1 and AKR1C3 showed higher expression in HCC samples, whereas CYP3A4, CA2, and TTR showed lower expression in HCC samples, as shown in Fig 7A. Thus, the five candidate genes showed differential expression patterns rather than uniform upregulation in HCC samples. ROC curves were plotted to assess the apparent discriminatory ability of each candidate gene within the same dataset. ROC analysis yielded AUC values of 0.913 for CYP3A4, 0.958 for KIFC1, 0.929 for AKR1C3, 0.902 for CA2, and 0.866 for TTR, as shown in Fig 7B. Because these ROC analyses were performed in the discovery dataset used for candidate gene prioritization, the AUC values should be interpreted as exploratory internal estimates and require validation in independent cohorts.

### Associations between candidate targets and estimated immune-cell fractions

**Fig 8:**
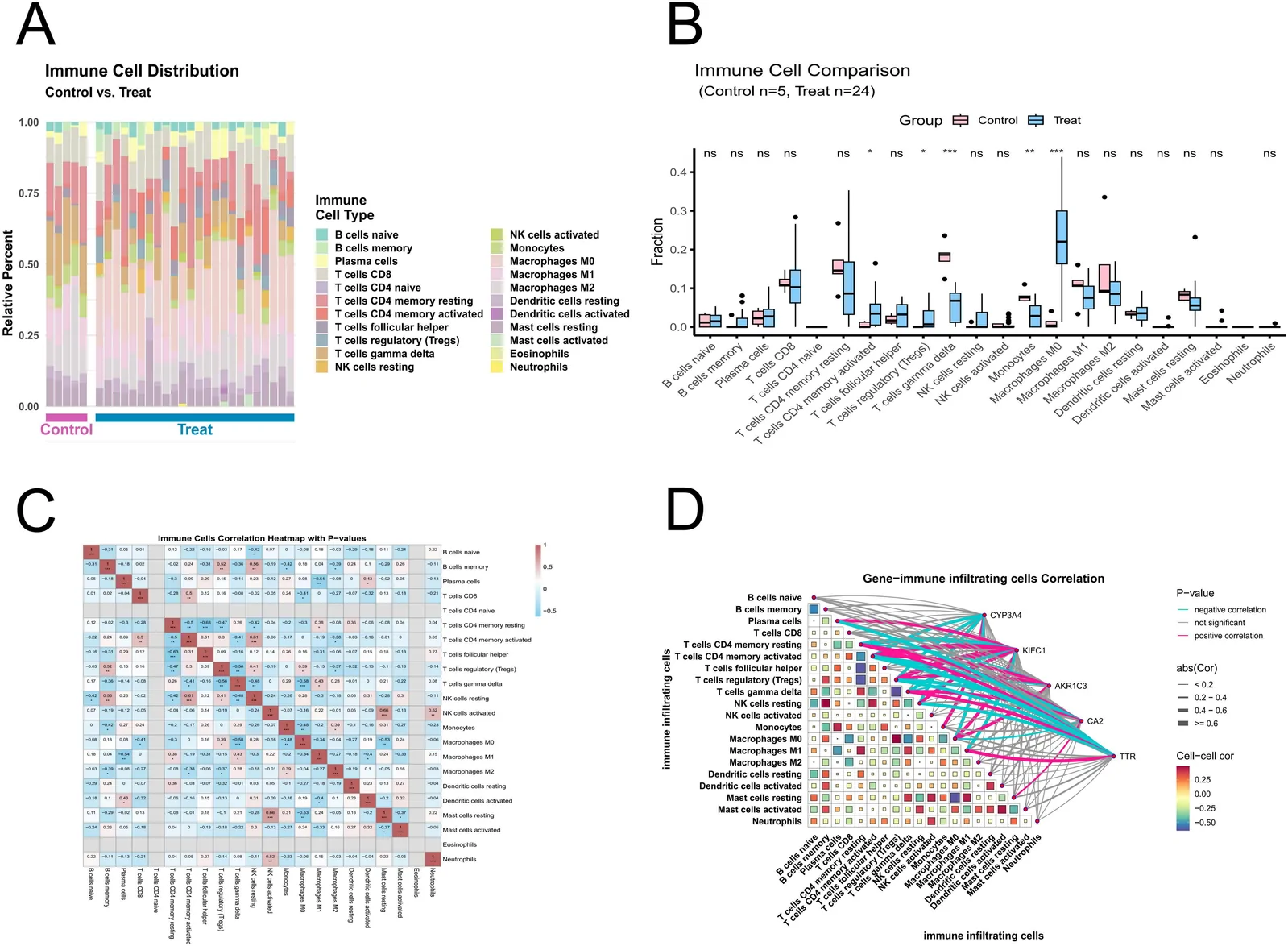
Associations between candidate targets and estimated immune-cell fractions. (A, B) CIBERSORT results for 22 immune-cell subtypes in HCC tissues versus control tissues. (C) Correlation analysis results among immune-cell subtypes in HCC. (D) Correlation analysis results between candidate target genes and estimated immune-cell fractions in HCC.

Based on the GSE36376 matrix file, CIBERSORT was used to estimate the relative fractions of 22 immune-cell subsets between the control and HCC groups. Fig 8A shows the relative proportion distribution of different immune cells in each sample of the two groups. Differential analysis showed higher estimated fractions of CD4 + memory activated T cells, regulatory T cells (Tregs), and macrophages M0 in the HCC group than in the control group, whereas T cells gamma delta and monocytes showed higher estimated fractions in control tissues, as shown in Fig 8B. Correlation analysis among immune cells showed: CD4^+^ memory activated T cells were positively correlated with resting NK cells and CD8^+^ T cells, and negatively correlated with follicular helper T cells and resting CD4^+^ memory T cells, etc. Regulatory T cells (Tregs) were positively correlated with macrophages M0, resting NK cells, etc., and negatively correlated with macrophages M2, T cells gamma delta, etc. Macrophages M0 were positively correlated with CD8^+^ T cells and negatively correlated with resting mast cells, monocytes, etc., as shown in Fig 8C. Further analysis of the correlation between the five candidate targets and estimated immune-cell fractions showed: CYP3A4 was positively correlated with monocytes and negatively correlated with CD4^+^ memory activated T cells, Tregs, etc. KIFC1 was positively correlated with plasma cells, CD4^+^ memory activated T cells, etc., and negatively correlated with resting CD4^+^ memory T cells, T cells gamma delta, etc. AKR1C3 was positively correlated with CD4^+^ memory activated T cells, Tregs, etc., and negatively correlated with resting CD4^+^ memory T cells, T cells gamma delta. CA2 was positively correlated with resting CD4^+^ memory T cells, T cells gamma delta, etc., and negatively correlated with CD4^+^ memory activated T cells, Tregs, etc. TTR was positively correlated with resting CD4^+^ memory T cells, T cells gamma delta, etc., and negatively correlated with plasma cells, CD4^+^ memory activated T cells, etc., as shown in Fig 8D.

### Molecular docking analysis of candidate targets and OTA

**Fig 9:**
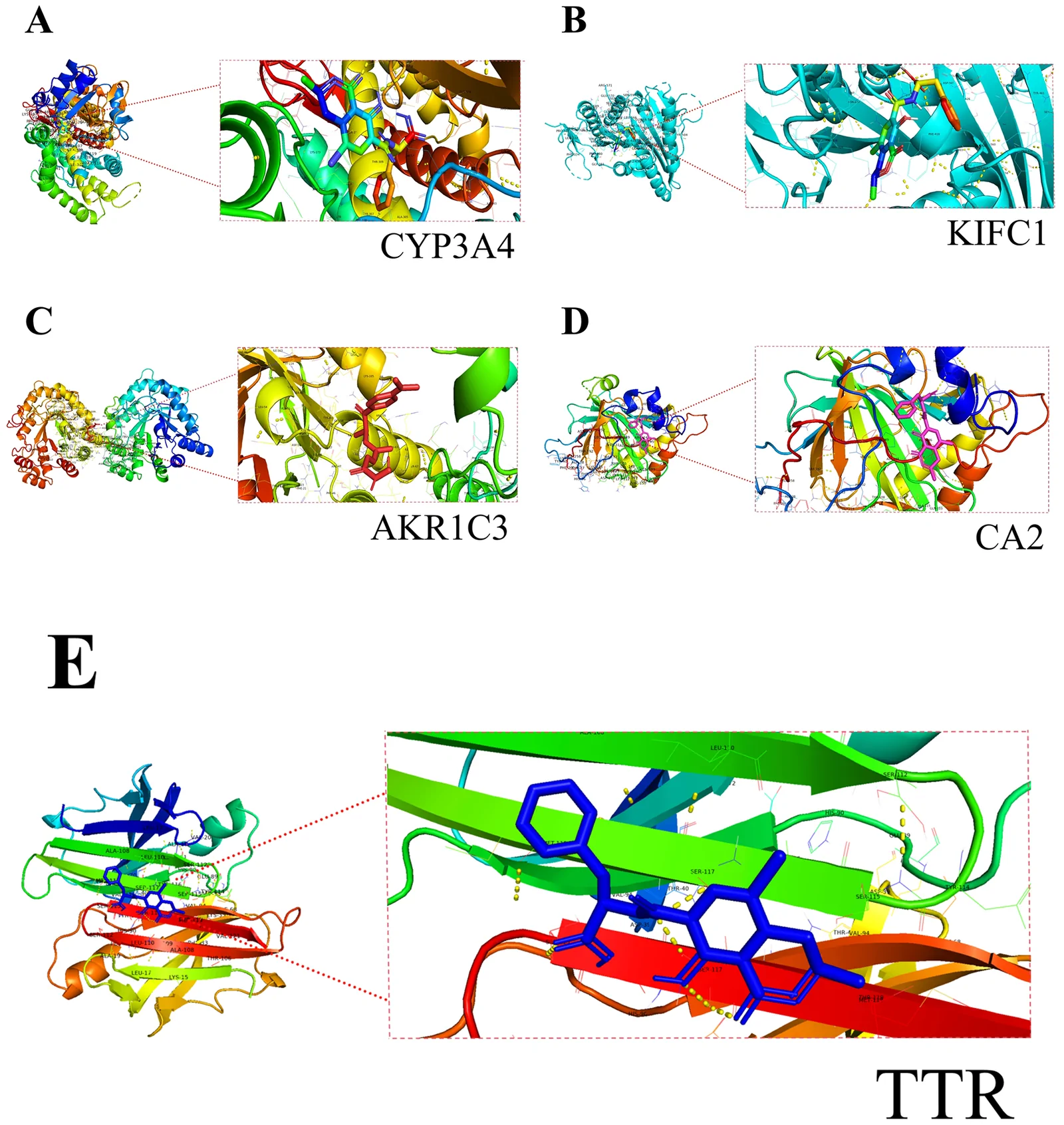
Predicted molecular docking poses of OTA with candidate targets. (A) Molecular docking of OTA and CYP3A4. (B) Molecular docking of OTA and KIFC1. (C) Molecular docking of OTA and AKR1C3. (D) Molecular docking of OTA and CA2. (E) Molecular docking of OTA and TTR.

Molecular docking was used to predict potential interactions between OTA and the prioritized candidate proteins CYP3A4, KIFC1, AKR1C3, CA2, and TTR. Lower docking energy scores were interpreted as indicating more favorable predicted binding under the selected docking conditions. The docking results are shown in Table 1. Among the evaluated candidate proteins, CYP3A4 showed the lowest predicted docking energy with OTA (−10.8 kcal/mol), followed by AKR1C3 (−10.3 kcal/mol), KIFC1 (−8.2 kcal/mol), TTR (−8.0 kcal/mol), and CA2 (−7.4 kcal/mol). These docking results were used for computational target prioritization and should not be interpreted as direct experimental evidence of binding. The docking poses were visualized using PyMOL, as shown in Fig 9.

**Table 1:** Predicted molecular docking energies for the top four OTA-target docking poses.

Compound	Molecular docking energy (kcal/mol)				
	CYP3A4	KIFC1	AKR1C3	CA2	TTR
Ochratoxin A	−10.8	−8.2	−10.3	−7.4	−8.0
	−9.8	−8.0	−8.5	−7.3	−7.8
	−9.7	−7.9	−8.5	−7.3	−7.7
	−9.4	−7.8	−8.4	−7.1	−7.7

### Molecular dynamics results

**Fig 10:**
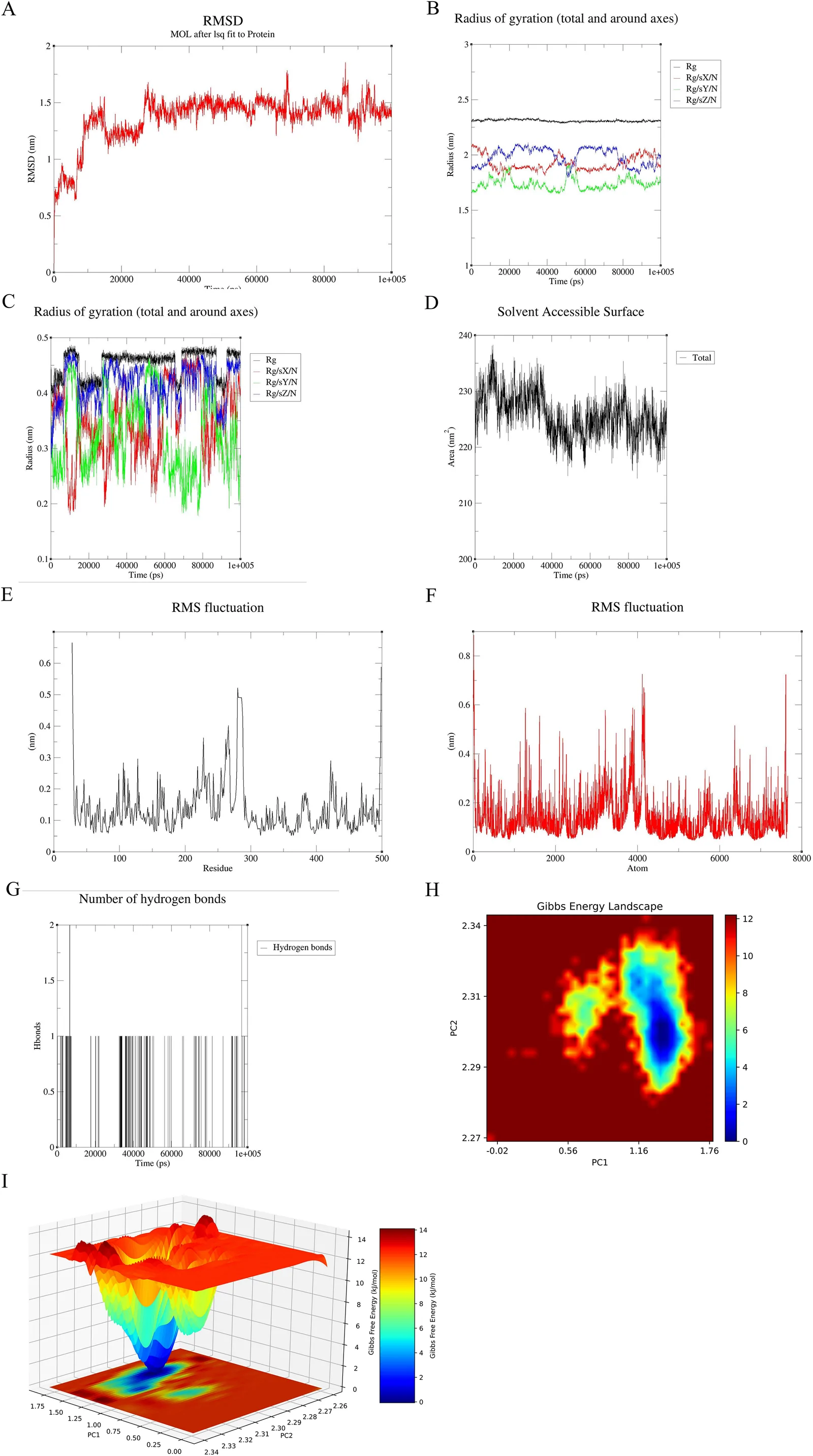
Molecular dynamics simulation results for the predicted CYP3A4-OTA complex. (A) Time evolution curve of ligand RMSD after fitting to protein. (B, C) Rg analysis derived from the CYP3A4-OTA complex trajectory. (D) SASA analysis. (E) Residue flexibility analysis. (F) Atom-level RMSF derived from the CYP3A4-OTA complex trajectory. (G) Number of hydrogen bonds between protein and ligand. (H) Two-dimensional Gibbs free energy landscape. (I) Three-dimensional Gibbs free energy landscape.

CYP3A4, which showed the most favorable predicted docking energy with OTA, was selected for molecular dynamics simulation. Because molecular docking provides only a static prediction, molecular dynamics simulation was used to explore the stability of the predicted CYP3A4-OTA complex under the selected simulation conditions. All quantitative analyses were derived from the receptor-ligand complex trajectory rather than from independent simulations of the receptor or ligand alone. RMSD analysis showed that the protein-fitted OTA RMSD increased during the early stage of the simulation and then remained within a relatively stable range, as shown in Fig 10A. Quantitative analysis of the equilibrated 20–100 ns trajectory showed a protein-fitted OTA RMSD of 1.435 ± 0.097 nm, complex Rg of 2.308 ± 0.010 nm, small-molecule Rg derived from the same complex trajectory of 0.457 ± 0.022 nm, SASA of 225.011 ± 3.502 nm², and an average hydrogen-bond number of 0.034 ± 0.184. The low average hydrogen-bond number suggests that the predicted stability of the CYP3A4-OTA complex was not mainly maintained by persistent hydrogen bonding. Residue-level and atom-level RMSF analyses showed limited local fluctuations in most regions, with residue-level RMSF of 0.134 ± 0.078 nm and atom-level RMSF of 0.158 ± 0.068 nm. The free-energy landscape showed that the simulated conformations were mainly distributed in low-energy regions, as shown in Fig 10H and 10I. Overall, these results provide computational support that the predicted CYP3A4-OTA complex remained relatively stable under the selected simulation conditions, but they do not demonstrate direct biochemical binding or functional consequences.

## Discussion

This study integrated network toxicology, machine learning, immune infiltration estimation, molecular docking, and molecular dynamics simulation to explore potential molecular associations between OTA exposure and HCC. Previous studies have described the toxicokinetics, toxicodynamics, molecular toxicity mechanisms, and carcinogenic relevance of OTA, while network-based system toxicology provides a useful framework for integrating compound-target-disease relationships [4–7]. In this context, the present study should be interpreted as a computational hypothesis-generating analysis rather than definitive mechanistic evidence. The identified genes and pathways provide candidate targets and testable hypotheses for future in vitro and in vivo validation.

The five prioritized candidate targets, CYP3A4, KIFC1, AKR1C3, CA2, and TTR, showed differential expression in the GSE36376 discovery dataset and apparent discriminatory ability within the same dataset. KIFC1 and AKR1C3 were upregulated in HCC samples, whereas CYP3A4, CA2, and TTR were downregulated. The revalidated expression patterns indicate that these five genes are differentially expressed rather than uniformly upregulated, and their expression trends are broadly consistent with those reported in previous studies related to hepatocellular carcinoma [10,12,16–18]. These results suggest that the five genes may be relevant to HCC-related transcriptional alterations and OTA-associated candidate mechanisms. However, because the same dataset was used for candidate gene prioritization and ROC assessment, the AUC values may overestimate discriminatory performance and should not be interpreted as independently validated diagnostic efficacy. Therefore, these genes should be regarded as prioritized candidate biomarkers rather than clinically validated diagnostic markers.

Among the prioritized candidates, CYP3A4 showed the most favorable predicted interaction with OTA in molecular docking and molecular dynamics analyses. CYP3A4 is an important hepatic cytochrome P450 enzyme involved in xenobiotic metabolism, and functional variation in CYP3A4 has been widely studied in relation to drug metabolism and pharmacogenetics [19–21]. Therefore, the predicted CYP3A4-OTA interaction supports CYP3A4 as a candidate target for future experimental testing. However, the present computational results do not confirm that CYP3A4 is a causal mediator of OTA-induced hepatotoxicity or hepatocarcinogenesis. Experimental binding assays and OTA exposure models are required to determine whether OTA directly affects CYP3A4 structure, activity, or downstream metabolic consequences.

The other prioritized targets may also be relevant to HCC-related biological processes. KIFC1 is involved in bipolar spindle formation and genomic stability, and has been proposed as a potential cancer-related therapeutic target [22,23]. AKR1C3 has been implicated in malignant transformation and carcinoma-related regulatory processes [24,25]. CA2 is related to carbonic anhydrase activity and may contribute to tumor microenvironment adaptation under acidic conditions [26,27]. TTR has been reported as a biomarker associated with prognosis in cancer-related contexts [28]. These published findings provide biological context for the prioritized genes, but they do not demonstrate that OTA directly regulates these targets. In particular, the observed differential expression of KIFC1, AKR1C3, CYP3A4, CA2, and TTR was derived from HCC versus control samples, not from OTA-treated experimental models.

Functional enrichment analysis suggested that OTA-HCC-associated candidate targets were involved in biological processes and pathways related to receptor signaling, cell adhesion, apoptosis, lipid metabolism, and cancer-related pathways. These pathway-level findings are consistent with the complex molecular landscape of HCC and with known OTA-related toxicity mechanisms [2,4–6]. Together, these results propose a testable computational model in which OTA-HCC-associated targets are linked to metabolic dysregulation, genomic-instability-related pathways, and immune-microenvironment remodeling. However, enrichment analysis cannot establish a temporal or causal sequence in which OTA first induces metabolic disorder, then genomic instability, and finally immune remodeling.

CIBERSORT-based immune infiltration analysis suggested differences in estimated immune-cell fractions between HCC and control samples and associations between candidate gene expression and immune-cell fractions. These findings raise the possibility that OTA-associated HCC may involve immune microenvironment remodeling. T-cell subsets, regulatory T-cell responses, and γδ T cells have been reported to participate in cancer immunity and liver cancer-related immune regulation [29,30]. Nevertheless, because CIBERSORT infers relative immune-cell fractions from bulk transcriptomic data, these associations require validation using experimental immune profiling, spatial methods, or single-cell approaches. The present results should therefore be interpreted as immune-related computational associations rather than direct evidence of immune-cell infiltration or functional immune suppression.

The 100 ns molecular dynamics simulation of the predicted CYP3A4-OTA complex provided additional computational support for the docking prediction. The RMSD, Rg, SASA, RMSF, hydrogen-bond, and free-energy landscape analyses derived from the receptor-ligand complex trajectory suggested that the predicted CYP3A4-OTA complex remained relatively stable under the selected simulation conditions. GROMACS-based molecular dynamics simulation is a useful computational approach for examining dynamic behavior of molecular systems [14,15]. However, molecular dynamics simulation does not demonstrate direct biochemical binding or functional consequences. Therefore, CYP3A4 should be considered a prioritized candidate target that requires further validation using binding assays, OTA exposure models, and functional experiments.

The primary contribution of this study lies in its integrated computational workflow, which combines target prediction, transcriptomic screening, candidate gene prioritization, immune-cell deconvolution, molecular docking, and molecular dynamics simulation. This workflow provides a structured framework for generating plausible hypotheses regarding OTA-associated HCC mechanisms. However, several limitations should be acknowledged. First, because this study was based on public databases and computational analyses, the results should be considered hypothesis-generating rather than causal evidence. Second, feature selection and ROC evaluation were performed using the same GSE36376 discovery dataset, which may overestimate discriminatory performance and limit generalizability. Independent external validation is required before the prioritized genes can be considered diagnostic biomarkers. Third, CIBERSORT infers relative immune-cell fractions from bulk transcriptomic data and cannot resolve spatial immune-cell organization, cell-cell interactions, or functional immune states. Fourth, molecular docking and molecular dynamics simulations support possible ligand-target interactions under computational conditions but do not demonstrate direct binding or functional consequences in biological systems. Redocking validation, reference ligand comparison, experimental binding assays, OTA exposure models, and target knockdown or overexpression experiments are needed to confirm the proposed mechanisms. Finally, only the CYP3A4-OTA complex was subjected to molecular dynamics simulation, and additional simulations of other candidate target complexes and independent replicate trajectories would be required to compare binding stability more comprehensively. Therefore, although the present workflow provides useful computational hypotheses, the proposed OTA-HCC-associated targets and pathways require further validation in independent datasets and experimental models.
